# Down-regulation of TRIB3 inhibits the progression of ovarian cancer via MEK/ERK signaling pathway

**DOI:** 10.1186/s12935-020-01509-z

**Published:** 2020-08-28

**Authors:** Shuang Wang, Caixia Wang, Xiao Li, Yuexin Hu, Rui Gou, Qian Guo, Xin Nie, Juanjuan Liu, Liancheng Zhu, Bei Lin

**Affiliations:** 1 Department of Obstetrics and Gynaecology, Shengjing Hospital Affiliated to China Medical University, No. 36, Sanhao Street, Heping District, Liaoning, 110004 China; 2 Key Laboratory of Maternal-Fetal Medicine of Liaoning Province, Key Laboratory of Obstetrics and Gynecology of Higher Education of Liaoning Province, Shenyang, Liaoning China

**Keywords:** TRIB3, Ovarian cancer, Prognosis, MEK/ERK, EMT

## Abstract

**Background:**

Tribbles pseudokinase 3 (TRIB3) protein is a pseudokinase which plays an important role in cellular stress, metabolism, and tumor progression. However, the expression and function of TRIB3 in ovarian cancer is unknown.

**Methods:**

TRIB3 expression was detected by immunohistochemistry in the ovarian tissue samples. Following down-regulation of TRIB3 by siRNA, multiple aspects of ovarian cancer cells were detected by the MTT assay, flow cytometry, scratch test and Transwell. Additionally, changes in related molecules and the MEK/ERK pathway were detected by western blotting. Finally, many bioinformatic methods, websites and databases, such as gene set enrichment analysis (GSEA), DVAID, Genemania, TISIDB and cBioPortal were used to study the TRIB3.

**Results:**

The expression level of TRIB3 was higher in ovarian epithelial malignant tumors as compared to other groups. Patients with a high expression level of TRIB3 had significantly shorter survival times,which was consistent with the results of analysis of the KM-plot database. Down-regulation of *TRIB3* expression significantly inhibited the proliferation, invasion, and migration capabilities of ovarian cancer cells, and induced apoptosis and cell cycle arrest. Following TRIB3 siRNA transfection, expression levels of relative proteins were found to be decreased. Additionally, analysis in DAVID website and GSEA revealed that TRIB3 expression was associated with multiple biological processes. Protein phosphorylation levels of MEK and ERK also decreased following TRIB3-siRNA transfection. The Genemania website was used to analyze the proteins that interact with TRIB3. Analysis of *TRIB3* in the TISIDB database and cBioPortal website showed that ovarian cancer patients with high levels of mutation in TRIB3 had poor prognosis, and that the expression of TRIB3 was related to immunomodulation.

**Conclusions:**

The TRIB3 was highly expressed and promoting the malignant behavior of ovarian cancer cells by activating the MEK-ERK signaling pathway. It was also found to be associated with genetic variations and immune modulators.

## Background

Among gynecological malignancies, ovarian cancer has the highest mortality rate, and poses serious threat to the health of women. The onset of the disease is insidious, and most patients who receive treatment are in the advanced stage of ovarian cancer. Although advancements in surgery and chemotherapy have improved the survival rate of ovarian cancer patients, post-operative recurrence and resistance to anti-cancer drugs have reduced the five-year survival rate of patients with advanced ovarian cancer to less than 50% [1, 2]. Therefore, it is significant to explore the mechanisms of ovarian cancer development and to identify tumor markers that may be effective therapeutic targets.

Tribbles pseudokinase 3 (TRIB3) belongs to the pseudokinase gene family [3]. It lacks ATP-specific binding sites and catalytic cores. It does not have kinase activity, but it can compete with kinases for peptide substrates and regulate the biological functions of kinases [4]. TRIB3 is an important stress gene that can be up-regulated by a variety of stimulating factors, and plays an important role in cell apoptosis, metabolism of blood glucose and lipid, and adipocyte differentiation [5]. Members of the TRIBs gene family, TRIB1 and TRIB2, are known oncogenes, and play an important role in the development of many tumors [6, 7]. Recent studies have shown that the expression of TRIB3 was found to be elevated in colorectal cancer [8], breast cancer [9, 10] and lung cancer [11], and was related to the poor prognosis. Only few studies have reported the role of TRIB3 expression in gynecological tumors, and these are limited to endometrial cancer. Felip et al. [12] found that ABTL0812 (an anti-cancer drug) inhibited the PI3K/AKT/mTORC1 axis by up-regulating the expression of TRIB3 to sensitize endometrial cancer cells. Qu et al. [13] found that the expression level of TRIB3 in endometrial cancer cells was higher than that in normal endometrial tissue. The overexpression of TRIB3 promoted endometrial cell apoptosis, while it inhibited cell proliferation, migration and invasion. However, the expression of TRIB3 and its effect on the malignant biological behavior of ovarian cancer has not been reported.

In the previous study, we used gene chip technology to show that the expression of TRIB3, over-expressed by human epididymis protein 4 (HE4), was significantly up-regulated in ovarian cancer cells. It is hypothesized that TRIB3 may be associated with the malignant behavior of ovarian cancer. To further explore the relationship between TRIB3 expression and the development of ovarian cancer, this study detected the expression of TRIB3 in ovarian tissue, and the effect of inhibition of TRIB3 expression on the malignant behavior of ovarian cancer cells. Simultaneously, multiple bioinformatics databases were used to objectively analyze the functions and mechanisms of action of TRIB3 in ovarian cancer progression.

## Materials and methods

### Tissue samples

From years 2008 to 2014, a total of 137 paraffin-embedded surgical pathological specimens were collected from the Department of Obstetrics and Gynecology, Shengjing Hospital, China Medical University. This consisted of 94 cases of ovarian epithelial malignant tumor (ovarian cancer group), 16 cases of epithelial ovarian borderline tumor (borderline group), 14 cases of benign ovarian tumor (benign group), and 13 cases of normal ovarian tissue (normal group). The average age of patients in the ovarian cancer group was 54.47 years (19–79 years), the borderline group was 50.47 years (19–84 years), the benign group was 50.71 years (28–78 years), and the normal group was 46.61 years (38 years-57 years). No statistically significant difference was observed between the groups (P > 0.05). In the ovarian cancer group, the pathological types were as follows: 46 cases of serous ovarian cancer, 10 cases of mucinous adenocarcinoma, 14 cases of endometrioid adenocarcinoma, 6 cases of clear cell carcinoma, and 18 cases of other pathological types. Pathological grading identified 23 highly differentiated tumors, 23 moderately differentiated tumors, and 48 poorly differentiated tumors. According to the staging criteria outlined by the International Federation of Obstetrics and Gynecology (FIGO, 2009), 22 cases were identified with FIGO stage I tumors, 14 cases with FIGO stage II tumors, 54 cases with FIGO stage III tumors, and 4 cases with FIGO stage IV tumors. There were 27 cases of lymph node metastasis, 60 cases without lymph node metastasis, and 7 cases without lymph node dissection. All cases were primary ovarian cancer patients with complete clinico-pathological data and no pre-operative chemoradiotherapy. The study was approved by the Research Ethics Committee of Shengjing Hospital, China Medical University.

### Immunohistochemistry and immunocytochemical staining

The specimens were fixed with 10% formalin, embedded in paraffin, and sliced to obtain 5-µm serial sections. Ultra-sensitive TM SP (mouse/rabbit) IHC kit (Maixin, China, Cat# KIT-9720) was used to detect the expression of TRIB3. A breast cancer paraffin section was used as the TRIB3 positive control, and phosphate buffered saline (PBS) was used as the negative control. Rabbit TRIB3 polyclonal antibody (Proteintech, Wuhan, China, Cat# 13300-1-AP) was used at a dilution of 1:1000. The presence of buffy granules in the cell membrane and cytoplasm was defined as positive. The dyeing intensity was recorded as 0 (no coloration), 1 (yellow), 2 (brownish-yellow), and 3 (brown). The percentage of stained cells throughout the section was scored as follows: 0 for < 5%, 1 for 5 to 25%, 2 for 26 to 50%, 3 for 51 to 75%, and 4 for > 75%. The final score was obtained by multiplying the intensity score and the percentage score. A final score of 0–2 indicated negative expression (−), 3–4 indicated weak positive expression (+), 5–8 indicated positive expression (++), and 9–12 indicated strong positive expression (+++). Each section was independently evaluated by two senior pathologists. If the specimen evaluation did not show consistency between the two pathologists, the sample was then evaluated by a third pathologist.

###  Cell culture and transfection

Ovarian cancer cells Caov3, OVCAR3, SKOV3 and A2780 were cultured in RPMI 1640 and ES-2 was McCoy’s 5A medium containing 10% FBS (Fetal bovine serum, Biological Industries, Israel) at 37 °C, 5% CO_2_ and saturated humidity. Caov3, OVCAR3, SKOV3 and ES-2 cell lines were purchased from the Cell Bank of the Chinese Academy of Sciences (Shanghai, China). A2780 was purchased from Jennio Biotech (Guangdong, China). The day before transfection, exponentially growing cells were seeded into 6-well plates. The confluence of cells during transfection was 40–60%. Before transfection, serum-free medium was added to each well. For transfection, lipofectamine 3000 (Thermo Fisher, Cat# L3000015) was diluted with 125 µl serum-free 1640 medium, and 5 µl of the diluted lipofectamine was added to each well. Following gentle mixing, it was incubated for 5 min at room temperature. TRIB3-siRNA (7 µl), or its negative control (see Table 1), was diluted with 250 µl of serum-free 1640 medium and mixed gently. The diluted TRIB3-siRNA or negative control was gently mixed with the diluted lipofectamine 3000, and incubated at room temperature for 10 min. The mixture was then added to the cells. Following transfection, the plate was incubated at 37 °C, in 5% CO2, for 8 to 12 h. The transfection medium was then replaced with RPMI 1640 or McCoy’s 5A containing 10% FBS. The cells were collected 48 h post transfection.

**Table 1:** TRIB3-siRNA and negative control siRNA

	5′–3′	5′–3′
TRIB3-homo-5	GGAGUUGGAUGACAACUUATT	UAAGUUGUCAUCCAACUCCTT
TRIB3-homo-7	GGUGUACCCCGUCCAGGAATT	UUCCUGGACGGGGUACACCTT
TRIB3-homo-11	GGACCUGAGAUACUCAGCUTT	AGCUGAGUAUCUCAGGUCCTT
Negative control	UUCUCCGAACGUGUCACGUTT	ACGUGACACGUUCGGAGAATT

### Western blot

After collecting the cells, chilled radioimmunoprecipitation assay (RIPA) buffer was added to the cell pellet, which was lysed by sonication in chilled conditions for 30 min. Following sonication, whole cell lysates were collected by centrifugation at 12,000 rpm for 30 min, at 4 °C. The bicinchoninic acid (BCA) method was used to determine the protein concentration of each lysate. Loading buffer (CWVBIO, China) was added to the samples, and the samples were denatured at 100 °C for 5 min. Proteins (80 µg) were separated using 10% sodium dodecyl sulphate-polyacrylamide gel electrophoresis (SDS-PAGE) (80 V concentrated gel, 120 V separation gel). The proteins were transferred to a polyvinylidene fluoride (PVDF) membrane and separated at 100V for 90 min, at 4 °C. The membrane was blocked using 5% skim milk, prepared with TBST, for 2 h at room temperature. The membrane was incubated with a primary antibody (1X tris-buffered saline (TBST) dilution) at 4 °C overnight. TRIB3 (Proteintech, rabbit polyclonal antibody, 1:1000, Cat# 13300-1-AP), E-cadherin (Proteintech, rabbit polyclonal antibody, 1: 2000, Cat# 20874-1-AP), N-cadherin (Proteintech, rabbit polyclonal antibody, 1:2000, Cat# 22018-1-AP), Vimentin (Proteintech, Rabbit polyclonal antibody, 1:2000, Cat# 10366-1-AP), Twist1 (Affinity, rabbit polyclonal antibody, 1:1000, Cat# AF4009), MMP2 (Proteintech, rabbit polyclonal antibody, 1:2000, Cat# 10373-2-AP), MMP9 (Proteintech, rabbit polyclonal antibody, 1:2000, Cat# 10375-2-AP), MEK (Affinity, rabbit polyclonal antibody, 1:1000, Cat# AF6384), p-MEK (Affinity, rabbit polyclonal antibody, 1:1000, Cat# AF8035), ERK (CST, rabbit polyclonal antibody, 1: 1000, Cat# 9102 s), p-ERK (CST, rabbit polyclonal antibody, 1:1000, Cat# 9101 s), GAPDH (ZSGB-BIO, murine monoclonal antibody, 1:2000, Cat# TA-08). On the following day, the membrane was washed thrice by shaking with 1X TBST for 5 min, and incubated with HRP-labeled goat anti-rabbit antibody or murine IgG (ZSGB-BIO, China, 1: 2000) for 2 h at room temperature, and subsequently washed as described above. Western chemiluminescence HRP substrate (Millipore, Billerica, MA, USA) was used to observe specific protein expression. The experiment was performed in triplicate.

### RT-PCR

The total RNA of the tissues was extracted by RNAiso Plus (Takara Bio, Inc., Shiga, Japan), and the purity and concentration of RNA were determined by UV spectrophotometer. The RT-PCR kit (TAKARA047A, Takara Bio, Inc., Shiga, Japan) of Super Script III First-Strand Synthesis System was used to reverse transcribe RNA into cDNA. The amplification conditions were: denaturation at 95 °C for 30 s, 95 °C for 5 s, and 60 °C for 30 s, for a total of 40 cycles (TAKARA820A, Takara Bio, Inc., Shiga, Japan). GAPDH primer: Forward: 5′-ACAACTTTGGTATCGTGGAAGG-3′, Reverse: 5′-GCCATCACGCCACAGTTTC-3′. TRIB3 primer: Forward: 5′-AAGAAGCGGTTGGAGTTGGATGAC-3′, Reverse: 5′-GTTGCACGATCTGGAGCAGTAGG-3′. Quantitative real-time PCR amplification was performed using the 7500Fast PCR instrument and the calculation was performed using the 2^−ΔΔCT^ method.

### Cell proliferation assay

Exponentially growing cells were prepared as a single cell suspension. Cells were seeded in 96-well plates (3000 cells/well) and assessed at time points of 0, 24, 48, 72 and 96 h. Time ‘0’ indicated 6 h post plating. Following addition of 20 µl of MTT solution (5 µg/ml, Cat#M8180, Solarbio, Beijing, China) to each well, the cells were incubated at 37 °C for 4 h. After removing the culture medium, 150 µl of dimethyl sulfoxide (DMSO, Solarbio, Beijing, China) was added to each well, and the plate was incubated with shaking for 5 min. Optical density (OD) was measured at 490 nm. The experiment was performed in triplicate.

### Cell apoptosis

The Annexin-V-fluorescein isothiocyanate (FITC)/propidium iodide (PI) (KGA107, KeyGen Biotech, Nanjing, China) double staining method was used to study apoptosis in ovarian cancer cells, following TRIB3-siRNA transfection. Each group had a blank control and a single staining control with two dyes. After the cells in each group were digested with trypsin without ethylenediaminetetraacetic acid (EDTA), the cells were centrifuged at 1000 rpm for 5 min to remove the supernatant. The cell pellet obtained was washed twice with PBS, and collected by centrifugation. The cells were resuspended in 500 µl of binding buffer provided in the apoptosis kit, and 5 µl of Annexin-V-FITC stain was added. Subsequently, 5 µl of PI stain was added. The cells were incubated in the dark for 15–20 min prior to detection by flow cytometry. The experiment was performed in triplicate.

### Cell cycle experiment

The cells in each group were collected, digested with trypsin without EDTA, washed twice with PBS, and centrifuged at 1000 rpm for 5 min, to adjust the cell concentration to 1 × 10^6^ cells/ml. After discarding the supernatant, 500 µl of 70% chilled ethanol was added at 4 °C, and the cells were fixed overnight. On the following day, ethanol was removed by centrifugation, and the cells were washed three times with cold solution of PBS. Subsequently, 500 µl of the PI/RNase (KGA512, KeyGen Biotech, Nanjing, China) staining working solution that was prepared beforehand (PI:RNase A was prepared at 9:1) was added. Following staining at 4 °C for 30 min, flow cytometry was performed. The experiment was performed in triplicate.

### Cell scratch test

Exponentially growing cells were prepared as a single cell suspension. Cells were seeded in 6-well plates. Once the cells reached 90% confluence, the plate was gently scraped with a 200 µl pipette tip in a straight line. Subsequently, the cells were washed twice with PBS and cultured in a serum-free medium. Scratch width was measured by microscope at 0 and 24 h. The experiment was conducted in triplicate.

### Transwell test

Matrigel was thawed at 4 °C overnight. A working solution was prepared by mixing Matrigel and ice-cold serum-free medium at a ratio of 1:8. Single-cell suspensions were prepared by digesting cells in the exponential growth stage. The upper chamber of the Transwell (Corning Coster) was filled with Matrigel working solution and dried overnight. Subsequently, 500 µl of medium containing 20% FBS was added to the lower chamber and 200 µl of the cell suspension (2 × 10^5^ cells/ml) in serum-free medium was added to the upper chamber. The Transwell was incubated at 37 °C for 48 h, washed thrice with PBS, and fixed with 4% paraformaldehyde for 30 min. Subsequently, the cells were washed thrice with PBS, and stained with crystal violet for 30 min. The upper surface of the cells was gently cleaned with a cotton swab and the ovarian cancer cells on the surface of the lower chamber were counted. The experiment was conducted in triplicate.

### Oncomine analysis

The Oncomine database (http://www.oncomine.org), which is currently the world’s largest oncogene chip database integrated with a data mining platform, was used to analyze the mRNA expression level of TRIB3 in the different cancer cell types.

### GEPIA dataset and Kaplan-Meier website analysis of tumor prognosis

GEPIA (http://gepia.cancer-pku.cn/), a database consisting of data retrieved from the UCSC Xena server, was used to analyze the differential expression of genes in tumor tissues and adjacent healthy tissues, in addition to estimation of patient survival and prognosis. Kaplan-Meier (http://kmplot.com), a website for the analysis of tumor prognosis, was used to analyze the prognostic value of TRIB3 in ovarian cancer. The conditions for screening were as follows: overall survival, follow-up for 60 months, and tissue type (in the case of serous carcinoma).

### Functional enrichment analysis using DAVID and GSEA

Functional and pathway enrichment analysis of genes co-expressed with TRIB3 were performed using DAVID (https://david.ncifcrf.gov), which integrates biological data and analysis tools to provide a systematic and comprehensive annotation of biological functions [14]. Genomic enrichment analysis was performed using the gene set enrichment analysis (GSEA) 3.0 software. The c2.cp.kegg.v6.1.symbols.gmt dataset was downloaded from the MsigDB database on the GSEA website. The default weighted enrichment analysis method was used to perform enrichment analysis on expression profile data and attribute files to high and low grouping. The random classification frequency was set to 1000.

### GeneMANIA analysis of interacting proteins

GeneMANIA (http://www.genemania.org) is a flexible web interface which constructs an interactive functional association network between genes with similar functions and query genes, to illustrate the relationship between genes and datasets. In this study, the database was used to construct a gene-gene interaction network for TRIB3 by including genes that are strongly associated with the family in terms of physical interactions, co-expression, prediction, co-localization, and genetic interactions. It shows TRIB3 as the central node, surrounded by 20 other nodes.

### cBioPortal website for genetic variation

The cBioPortal website (http://www.cbioportal.org) is an open-access, open-source resource containing data retrieved from the TCGA database, for the interactive exploration of multiple cancer genomics datasets [15]. Analysis of genetic variation of TRIB3 using cBioPortal database(489 cases from TCGA, Nature 2011; 585 cases from TCGA, PanCancer Atlas; and 606 cases from TCGA, Provisional).

### Correlation analysis with immune modulators on TISIDB

The TISIDB database (http://cis.hku.hk/TISIDB) integrates data retrieved from seven public databases that include data of genes associated with anti-tumor immunity, high-throughput screening data of genes associated with resistance or sensitivity of tumor cells to T cell-mediated immunotherapy, mass spectrometric analysis, and multi-omics data of immune cells adjacent to the tumor cells [16].Through database mining and high-throughput data analysis, the gene of interest can be queried for tumor-immune interactions, which can generate testable hypotheses and high-quality data.

### Statistical analysis

The SPSS 21.0 statistical software (IBM Corporation, Armonk, NY, USA) was used to analyze the data, and the GraphPad Prism 8.0 software was used to draw graphics. The results obtained have been expressed as mean plus or minus standard deviation. The statistical difference between the two groups was analyzed by Student’s t-test, and one-way analysis of variance (ANOVA) was used to compare the two groups. Kaplan-Meier curves were used for survival analysis. Two-tailed P < 0.05 indicates a statistically significant result. *P < 0.05; **P < 0.01; ***P < 0.001.

## Results

### TRIB3 expression in different ovarian tissues and its clinical significance

**Fig. 1:**
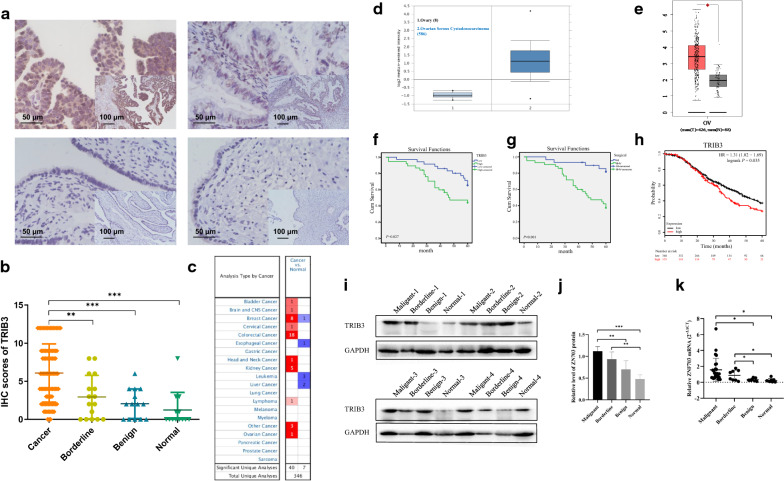
High TRIB3 expression in patients with ovarian cancer associated with poor prognosis. **a** TRIB3 expression in ovarian tissues samples (× 400, lower right × 100). **b** Immunostaining scores of TRIB3 in ovarian tissues samples. **c**, **d** TRIB3 mRNA expression in the various tumors from Oncomine database. **e** TRIB3 mRNA expression in the GEPAI database. **f**, **g** Overall survival analysis according to TRIB3 expression and FIGO stage. TRIB3 mRNA expression in ovarian cancer in Oncomine database. **h** TRIB3 expression with overall survival (OS) in Kaplan-Meier Plotter. **i** The expression of TRIB3 protein in ovarian tissues by western blot (n = 04 for each group). **j** The relative gray values of TRIB3 protein in ovarian tissues in **i**. **k** TRIB3 mRNA expression in ovarian tissues by qRT-PCR (n = 26 for malignant group, n = 08 for borderline group, benign group and normal group). For western blot, GAPDH was used as an internal control. Data are presented as mean ± SD. *P < 0.05; *P < 0.01; ***P < 0.001

The TRIB3 protein was expressed both in the cytoplasm and in the nucleus, but primary expression was observed in the nucleus (Fig. 1a). The positive expression level of TRIB3 was higher (75.7%) in the ovarian cancer group as compared to the level observed in the borderline group (50%) (*P* > 0.05). However, when compared to the benign group (35.7%) and normal ovarian tissue groups (23.1%), the positive expression level of TRIB3 was significantly higher in the ovarian cancer group (75.7%) (*P* < 0.01). The degree of TRIB3 high positive expression in the ovarian cancer group (60.6%) was significantly higher than that in the borderline group (25%), benign group (7.1%), and normal ovarian tissue groups (0%). The degree of the high positive expression and the positive expression level of TRIB3 were found to be higher in the borderline group as compared to the levels observed in the benign group and the normal group (*P* > 0.05) (Table 2) (Fig. 1b). Study of TRIB3 expression in Oncomine database showed that TRIB3 was highly expressed in the many cancer group compared to the normal tissue group (Fig. 1c), for ovarian cancer ,TRIB3 was significantly highly expressed in 586 ovarian serous cyst adenocarcinomas compared with 8 normal ovarian tissues (Fig. 1d). Consistent results were obtained during the analysis of TRIB3 expression in 426 cases of ovarian cancer and 88 individuals with normal ovarian tissue, in the GEPAI database (Fig. 1e).

**Table 2:** Expression of TRIB3 in different types of ovarian tissue

Group	Cases	Low		High		Positive rate (%)	High Positive rate (%)
		−	+	++	+++		
Malignant	94	23	19	23	29	75.5^a,b^	60.6^c,d^
Borderline	16	8	4	2	2	50^e,f^	25^ g,h^
Benign	14	9	4	1	0	35.7	7.1
Normal	13	10	3	0	0	23.1	0

^a^Malignant vs. benign (**P = 0.004)

^b^Malignant vs. normal (***P < 0.001)

^c^Malignant vs. benign (***P = 0.001)

^d^Malignant vs. normal (***P < 0.001)

^e^Borderline vs. benign (*P* = 0.431)

^f^Borderline vs. normal (*P* = 0.249)

^g^Borderline vs. benign (*P* = 0.336)

^h^Borderline vs. normal (*P* = 0.107)

This study evaluated a total of 94 ovarian cancer specimens. The positive expression level of TRIB3 (79.3%) in the late FIGO group (stage III to stage IV) was found to be higher than that in the early FIGO group (stage I to stage II) (69.4%) (*P* > 0.05). In patients with advanced ovarian cancer, the extent of TRIB3 expression was found to be 58.6%, that was higher than that observed in patients with early stage of the disease (50%) (*P* > 0.05). Additionally, the analysis found no significant difference in the extent of expression and positive expression of TRIB3 with the degree of differentiation, lymph node metastasis, and pathological types (*P* > 0.05) (Table 3).

**Table 3:** Relationship between TRIB3 expression and clinicopathological parameters of ovarian epithelial malignant tumors

Groups	Cases	Low		High		Positive rate (%)	*P*-value	High expression rate (%)	*P*-value
		(−)	(+)	(++)	(+++)				
FIGO stage									
I–II	36	11	7	9	9	69.4	*P *= 0.279	50.0	*P *= 0.414
III–IV	58	12	12	14	20	79.3		58.6	
Differentiation									
Well-moderate	46	9	9	14	14	80.4	*P *= 0.279	60.9	*P *= 0.289
Poor	48	14	10	9	15	70.8		50	
Lymphatic metastasis									
No	60	16	9	18	17	73.3	*P *= 0.540	58.3	*P *= 0.229
Yes	27	6	9	3	9	79.3		41.4	
Unknown^a^	7	1	1	2	3	85.7		71.4	
Pathological type									
Serous	46	10	12	10	14	78.3	*P *= 0.371	52.2	*P *= 0.786
Mucinous	10	4	1	2	3	60		50	
Endometrioid	14	3	2	4	5	78.6		64.3	
Clear cell carcinoma	6	3	1	1	1	50		33.3	
Poorly differentiated adenocarcinoma	18	3	3	6	6	83.3		66.	

^a^ 7 patients without lymphadenectomy

Follow-up of patients with ovarian cancer was performed until the 30th of April, 2019. The mean survival time in the TRIB3 positive- and negative-expression groups was 50.4 months and 62.5 months, respectively. Kaplan-Meier analysis showed that TRIB3 positive expression was associated with shortened overall survival of the patients (*P* = 0.027) (Fig. 1f), and FIGO stages I–II and stages III–IV were associated with poor prognosis (*P* < 0.05) (Fig. 1g). KM-plot website analysis showed that serous ovarian cancer patients with high expression levels of TRIB3 had significantly shorter survival times compared to patients with low expression levels of TRIB3 (*P* = 0.0035) (Fig. 1h).

Univariate Cox regression analysis suggested that TRIB3 expression, the FIGO stage of the cancer, and lymph node metastasis were risk factors for the prognosis of ovarian cancer. Multivariate Cox regression analysis indicated that the FIGO stage was an independent risk factor that affected the survival time of ovarian cancer patients (Table 4).

**Table 4:** Univariate and multivariate cox analysis of different clinicopathological parameters with ovarian cancer

Variable	Categories	Univariate analysis		*P*	Multivariate analysis		*P*
		HR	95% CI		HR	95% CI	
Age	≤ 54	1.891	(0.911–3.926)	0.087			
	> 54						
Differentiation	Well-moderate	1.712	(0.838–3.496)	0.094			
	Poor						
FIGO stage	I–II	4.980	(1.907–13.005)	0.001**	3.403	(1.119–10.346)	0.031^*^
	III–IV						
Lymph node metastasis	No	2.817	(1.300–6.105)	0.009**	1.466	(0.619–3.471)	0.384
	Yes						
TRIB3	Low	2.194	(1.068–4.507)	0.032*	1.517	(0.682–3.374)	0.306
	High						

For validation of the data from online websites, we performed western blot and qRT-PCR of ovarian tissues. The analysis revealed that the TRIB3 protein and mRNA were both higher in ovarian cancer tissues than benign and normal ovarian tissues (*P* < 0.05) (Fig. 1i–k). They were consistent with the results from Immunohistochemistry and on websites.

### TRIB3 down-regulation inhibited ovarian cancer cell proliferation, induced apoptosis and cell cycle arrest in the G0/G1 phase

**Fig. 2:**
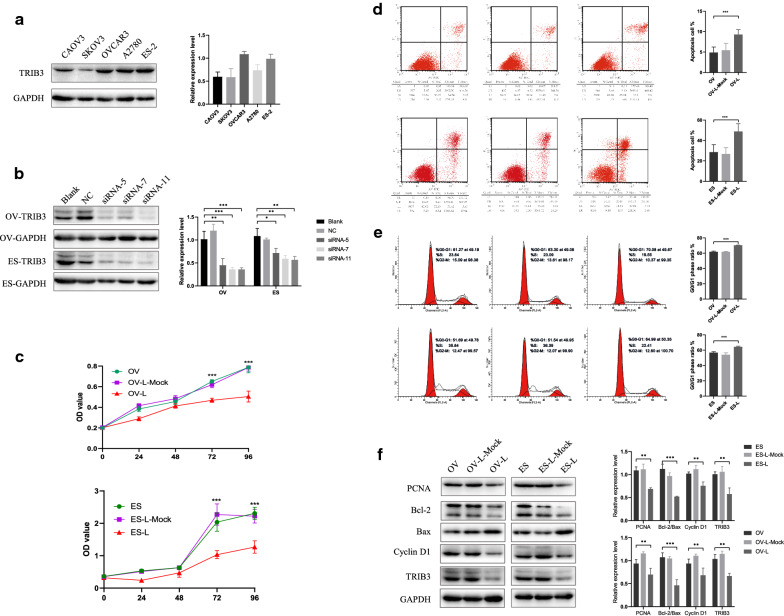
TRIB3-siRNA inhibited proliferation and induced apoptosis and cell cycle arrest in ovarian cancer cells. **a** The expression of TRIB3 protein in five OC cell lines using western blot. **b** Downregulation of TRIB3 protein using western blot. **c** TRIB3-siRNA inhibited cell proliferation of ovarian cancer cells. **d** TRIB3-siRNA increased apoptosis of ovarian cancer cells. **e** G0/G1 phase arrest of ovarian cancer cells after TRIB3 siRNA transfection. **f** Western blot analysis showed that the expression of PCNA, Bcl2/Bax, cyclin D1 protein in ovarian cancer cells decreased after transfection with TRIB3-siRNA. For western blot, GAPDH was used as an internal control. Data are presented as mean ± SD. *P < 0.05; **P < 0.01; ***P < 0.001

To assess the effects of TRIB3 expression on the proliferation, apoptosis, and cell cycle of ovarian cancer cells, the expression level of TRIB3 in five ovarian cancer cell lines (Caov3, OVCAR3, SKOV3, ES-2, and A2780 cells) was examined (Fig. 2a). The expression level of TRIB3 was higher in OVCAR3 and in ES-2 as compared to the levels in other cell lines. SiRNA was used to down-regulate the expression of TRIB3 in the OVCAR3 and in ES-2 cell lines (Fig. 2b). Cell proliferation, cell cycle and apoptotic processes were studied in these cell lines before and after the down-regulation of TRIB3. MTT assay results showed that the cell proliferation ability was remarkably slower (*P* < 0.05) as compared to that in the control group (Fig. 2c), and apoptosis was clearly elevated (*P* < 0.05) following transfection with TRIB3-siRNA (Fig. 2d). Flow cytometry confirmed that the percentage of cells in the G0/G1 phase was significantly increased (*P* < 0.05), and the proportion of S and G2-M phase cells was significantly reduced (*P* < 0.05) following TRIB3 down-regulation, as compared to that in the control group (Fig. 2e). Western blot was used to detect proliferation-related indicators like PCNA, apoptosis-related indicators like Bcl2 and Bax, and cell cycle-related indicators like cyclin D1. It was found that, following TRIB3-siRNA transfection, the expression of PCNA, Bcl2, and cyclin D1 decreased, while that of Bax increased (Fig. 2f). These results suggest that the down-regulation of TRIB3 could inhibit cell proliferation, arrest the cell cycle at the G0/G1 phase, and promote apoptosis.

### TRIB3 down-regulation inhibits invasion, migration and epithelial mesenchymal transition (EMT) of ovarian cancer cells

**Fig. 3:**
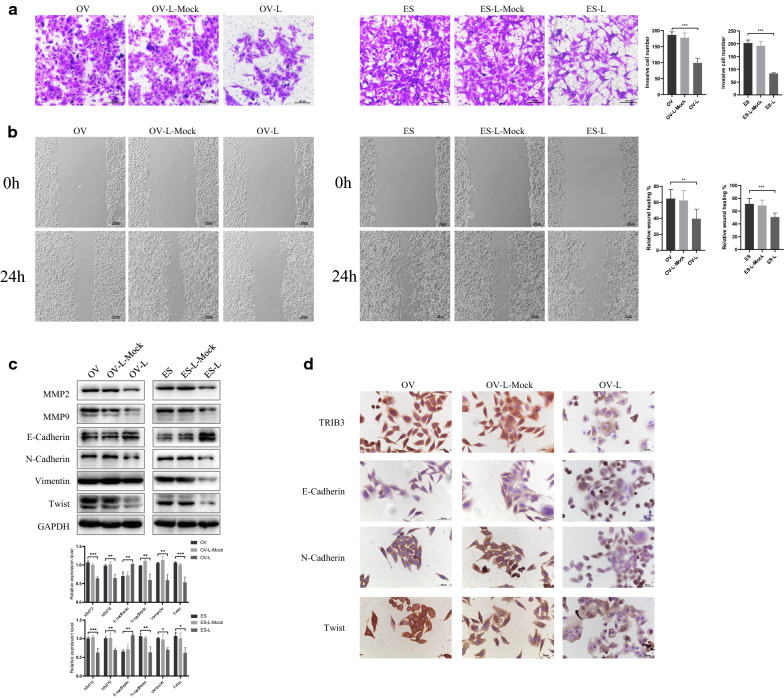
TRIB3-siRNA inhibited invasion, migration and epithelial mesenchymal transition (EMT) of ovarian cancer cells. **a** TRIB3-siRNA suppressed invasion of ovarian cancer cells (×200). **b** TRIB3-siRNA inhibited migration of ovarian cancer cells (×100). **c** Transfection of TRIB3 siRNA decreased the expression of MMP2, MMP9, E-cadherin, N-cadherin, Vimentin, Twist protein. GAPDH was used as an internal control. **d** Immunocytochemical staining of TRIB3, E-cadherin, N-cadherin, Twist in OVCAR3 cell. Data are presented as mean ± SD. *P < 0.05; **P < 0.01; ***P < 0.001

In order to further explore the effect of TRIB3 down-regulation on the invasion and migration of ovarian cancer cells, the Transwell was used to measure the changes in cell invasion of these two cell lines. It was found that the invasiveness and migration of OVCAR3 and ES-2 cells significantly reduced following the down-regulation of TRIB3 (*P* < 0.05) (Fig. 3a, b). Western blot analysis showed that the protein expression levels of MMP2 and MMP9 were also decreased (Fig. 3c). These data indicate that the down-regulation of TRIB3 could inhibit the invasion and migration of ovarian cancer cells. Western blot was performed to measure the expression of EMT-related molecules like E-cadherin, N-cadherin, and Vimentin, Twist in OVCAR3 and in ES-2 cells, before and after TRIB3-siRNA transfection. The results showed that, following the down-regulation of TRIB3, the expression of E-cadherin increased, while that of N-cadherin, Vimentin and Twist decreased, as shown by western blot(*P* < 0.05). And immunocytochemical staining assays was consistent with it (Fig. 3d). These results indicate that the down-regulation of TRIB3 could inhibit the EMT process of ovarian cancer cells.

### TRIB3 down-regulation activated the MEK-ERK signaling pathway

**Fig. 4:**
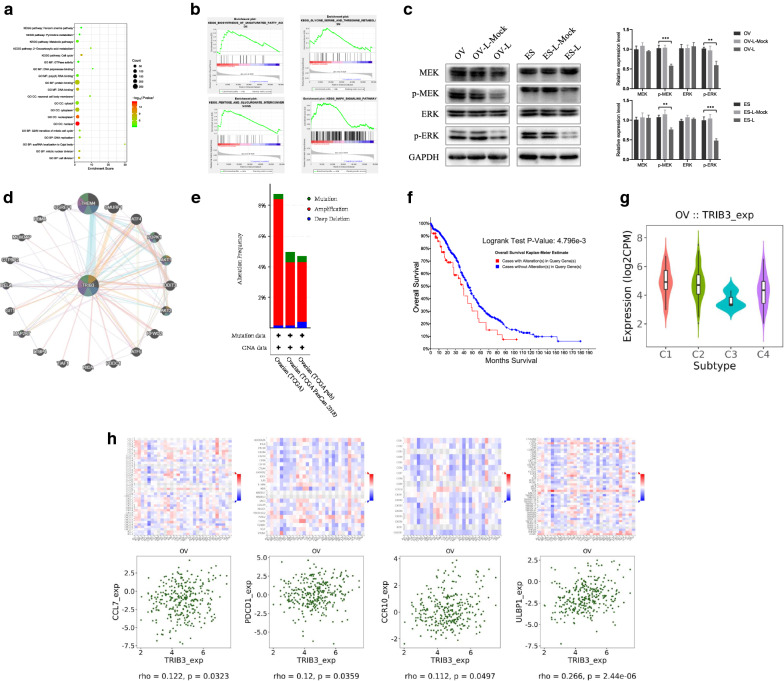
The mechanisms of TRIB3 function in ovarian cancer. **a** The bubble plot of top 20 biological functions and pathways related to TRIB3. **b** GSEA analysis of TRIB3-reated enrichment gene sets. **c** Western blot analysis showed that the expression of MEK, p-MEK, ERK, p-ERK protein in ovarian cancer cells decreased after transfection with TRIB3-siRNA. GAPDH was used as an internal control. **d** The gene interaction network of top 20 genes related to TRIB3 in Genemania. **e** TRIB3 genetic variation analysis in cBioPortal. **f** Survival analysis of TRIB3 genetic variation in cBioPortal. **g**, **h** The relationship between TRIB3 and immune sub-types or molecular sub-types in the TISIDB database. Data are presented as mean ± SD. *P < 0.05; **P < 0.01; ***P < 0.001

Functional and pathway enrichment analysis of TRIB3-related genes in the protein-protein interaction (PPI) network was performed using the DAVID database. The “ggplot2” R software package was used to view the top 20 biological functions and pathways related to TRIB3. These genes were found to be involved in multiple biological processes like metabolic pathways, protein binding, cell division, and cell cycle (Fig. 4a). GSEA showed that cells with high level of TRIB3 expression had augmented cell metabolism and MAPK signaling pathways (Fig. 4b).

To further investigate the mechanism of TRIB3, western blot was used to detect changes in MEK and ERK protein phosphorylation levels in OVCAR3 and in ES-2 cells, before and after TRIB3 down-regulation. The results showed that, following TRIB3 down-regulation, the expression of p-MEK and p-ERK decreased *(P* < 0.05) in both cell lines, while protein levels of MEK and ERK showed no significant variation (*P* > 0.05) (Fig. 4c). These results indicate that TRIB3 down-regulation may activate the MEK-ERK signaling pathway.

### Genemania website constructed TRIB3 gene-gene interaction network

The top 20 genes related to TRIB3 were shown in the gene interaction network (Fig. 4d). The top 5 most relevant genes include THEM4 (thioesterase superfamily member 4), SMURF1 (SMAD-specific E3 ubiquitin protein ligase 1), ATF4 (Activating transcription factor 4), PDPK1 (3-phosphate inositol-dependent protein kinase 1) and AKT1 (AKT serine/threonine kinase 1), of which THEM4, PDPK1, and AKT1 were related to TRIB3 in terms of pathways, and SMURF1 was predicted. ATF4 physically interacted with TRIB3. Further functional analysis revealed that these genes showed the greatest association with the Fc receptor signaling pathway (FDR = 3.24 × 10^− 4^). Moreover, it was also related to the metabolism of insulin, glucose, and fat, and cell surface receptor signaling pathways which regulate immune response.

### cBioPortal for TRIB3 genetic variation analysis

A total of 1,680 ovarian cancer cases were analyzed for genetic changes in TRIB3, in the cBioPortal website (Fig. 4e). Overall, the genetic changes in TRIB3 were found to be less than 10%, of which TCGA (Nature, 2011) was found to be 8.75% (the incidence of amplification, deep deletion, and mutation were 8.23%, 0.17%, and 0.34%, respectively), TCGA (PanCancer Atlas) was found to be 4.97% (the incidence of amplification, deep deletion and mutation was 4.11%, 0.17%, and 0.68%, respectively), and TCGA (Provisional) was found to be 4.7% (the incidence of amplification, deep deletion and mutation was 3.89%, 0.41%, and 0.41%, respectively). Survival analysis revealed significantly short survival times in ovarian cancer patients with altered TRIB3 gene (*P* = 4.796 × 10^− 03^) (Fig. 4f).

### The correlation between TRIB3 expression and immunity

When analyzing the relationship between TRIB3 expression and immune sub-types through the TISIDB database, it was found that the expression of TRIB3 was lowest in the C3 sub-type and highest in the C1 sub-type, indicating that TRIB3 expression may result in poor prognosis (Fig. 4g). The TISIDB database was used to analyze the expression of TRIB3 in the molecular sub-types of ovarian cancer (Fig. 4h). With regards to chemokines, the expression of TRIB3 was found to positively correlate with CC7 (rho = 0.122,*P* = 0.0323). Analysis of the relationship between TRIB3 expression and immune inhibitors revealed that the expression of TRIB3 positively correlated with PD1 (rho =  0,12, *P* = 0.0359), and CC10 (rho =  0.112, *P* = 0.0497). In the context of receptors, our study found that TRIB3 expression positively correlated with ULBP1 (rho =  0.266, *P* = 2.44 × 10^− 06^). These results suggest that TRIB3 expression may be involved in regulating the aforementioned immune molecules.

## Discussion

Currently, ovarian cancer represents the gynecological malignant tumor with the highest mortality rate. Owing to the lack of typical clinical symptoms, signs and detection methods in the early stage, many patients advanced stage at the time of consultation, with short survival rates and poor prognosis [2]. Moreover, the mechanisms leading to malignancy in ovarian cancer, remain poorly characterized.

The TRIB3 protein belongs to the family of pseudokinases. This family of proteins have kinase-like domains, but lack kinase activity owing to the lack of specific ATP binding sites and catalytic core [17]. Under physiological conditions, TRIB3 expression regulates a series of processes like cell stress response, cell proliferation and differentiation, glucose and lipid metabolism, and can respond to a variety of stresses, making it a “pressure regulator” switch for homeostasis, metabolic diseases and cancers [18–21]. Abnormal expression of TRIB3 protein causes liver fibrosis [22], suggesting that the normal expression of TRIB3 protein plays an important role in various physiological and pathological processes in human tissues and organs.

Recently, the role of TRIB3 expression in tumors has gradually attracted widespread attention from researchers. Existing studies have confirmed that the expression of TRIB3 is increased in solid tumors such as colorectal cancer, melanoma, breast cancer, liver cancer, lung cancer, and leukemia, thereby making it a marker of cancer [8, 9, 11, 20, 21, 23]. In the present study, through immunohistochemical experiments, western blot and qRT-PCR, we identified that TRIB3 was highly expressed in ovarian cancer in protein and mRNA level, which was confirmed by expression analysis studies in the Oncomine and GEPAI databases. Although our analysis revealed that there was no significant difference between TRIB3 expression and FIGO stage of the cancer, pathological type, degree of differentiation, and lymph node metastasis, we hypothesize that this may be due to the small sample size. However, the Kaplan-Meier survival analysis showed that the high expression level of TRIB3 in ovarian cancer cells was significantly associated with short survival rates and poor prognosis, as supported by the analysis in the KM-plot website. The above results suggest that TRIB3 expression may be an indicator for evaluating the prognosis of ovarian cancer patients. Studies reported that knocking out TRIB3 inhibited tumor formation and cancer progression, disrupting TRIB3–SQSTM1 or TRIB3–PML/RARα interactions through specific peptides, which is a potential strategy for treatment of certain solid cancers and acute promyelocytic leukemia [19–21]. TRIB3 expression was also found to be associated with tumor metabolism, as revealed by the induction of mutual antagonism between autophagy and ubiquitin proteasome system (UPS) [24, 25]. Previous studies have reported that TRIB3 is a potential target for cancer treatment [23]. However, the function of TRIB3 in gynecological malignancies has been rarely studied. In this study, following inhibition of TRIB3 expression in the ovarian cancer cell lines OVCAR3 and ES-2, it was found that the proliferation, migration, and invasion, of the ovarian cancer cells decreased, apoptosis increased, and cell cycle arrest occurred at the G0/G1 phase. This shows that TRIB3 expression has a cancer-promoting role in ovarian cancer. In esophageal squamous cell carcinoma (ESCC), upregulation of TRIB3 conferred radio-resistance by β-TrCP-mediated TAZ ubiquitination and degradation and interaction with TAZ in vitro and in vivo [26]. In breast cancer, TRIB3 expression positively correlates with the spheroid formation in vitro and tumor engraftment efficiency in vivo [27]. Furthermore, Julia Izrailit et al. [28] also proved the importance of TRIB3 for tumor growth in breast cancer in vitro and in vivo. They also found TRIB3 was a major regulator of Notch through the MAPK-ERK and TGFβ pathways [28]. But our work was only performed in vitro, which would be a weakness of our research and our future plans.

To further explore the mechanism by which TRIB3 expression affected the malignant behavior of ovarian cancer cells, the functional and pathway enrichment analysis of TRIB3 was performed using DAVID. The results were consistent with previously published studies, suggesting that TRIB3 expression plays a role in glucose and lipid metabolism. Additionally, our study focused on the MAPK signaling pathway that was enriched. MAPK is a serine/threonine protein kinase which widely occurs in cells and plays an important role in a variety of signal transduction processes in mammalian cells. p38 MAPK is closely associated with apoptosis and cell cycle changes in many malignant tumors. In in human bone marrow-derived mesenchymal stem cells (hBMSCs), TRIB3, regulated by FAK in a way that depends on PI3K/AKT, played an vital role in proliferation and osteogenic differentiation by modulating ERK1/2 activity at the middle stage of differentiation [29]. In renal cell carcinoma (RCC), researchers have found that high expression of TRIB3, which is induced by HIF-1α, enhanced cell proliferation, migration and invasion abilities by regulating the MAPK pathway [30]. And we found that there was a reduction in the phosphorylation of MEK proteins and ERK proteins in ovarian cancer cell lines following inhibition of TRIB3 expression. This was the first study to confirm that TRIB3 down-regulation may activate the MEK/ERK signaling pathway in ovarian cancer cells.

In EMT, polarized epithelial cells lose their adhesion capabilities, and acquire the phenotype of mesenchymal cells, along with their characteristics of migration and invasion [31]. This study revealed that the EMT process was inhibited following inhibition of TRIB3 expression in ovarian cancer cell lines. Additionally, the MAPK signaling pathway can also regulate EMT by stabilizing the TWIST1 protein [32]. This is consistent with the suppression of the MEK/ERK signaling pathway by the inhibition of TRIB3 expression. In a study involving leukemia, researchers confirmed that TRIB3 protein could bind to the WR domain of TWIST1 and contribute to its stability, by inhibiting its ubiquitination. By using peptides to target the TWIST1 WR domain, the TRIB3/TWIST1 interaction could be disrupted, thereby specifically inhibiting APL cell survival [21]. This was consistent with the experimental results of this study. Consequently, the impact of TRIB3 expression on EMT is worthy of recognition.

Many proteins have been reported to interact with TRIB3. The TRIB3 protein can bind to a variety of kinase-dependent proteins and regulate their functions by regulating their phosphorylation, thereby aiding in the signal transduction of multiple pathways [33]. The TRIB3 protein structure is inseparable from the structure of pseudokinase. Further analysis through the Genemania’s website revealed that TRIB3 interacts with proteins involved in co-localization, physical binding, and certain interacting pathways. However, this requires further experimental verification. It has been reported that TRIB3 may determine its own degradation by binding to the E3 ubiquitin ligase SIAH1 [34]. Through this property, TRIB3 could promote the ubiquitination and degradation of different cell cycle regulatory proteins to achieve cell cycle regulation [35].

The occurrence and development of tumors are closely related to genetic changes. According to the cBioPortal database, it was found that the genetic changes in TRIB3 was less than 10% as compared to the genetic changes of other genes involved in ovarian cancers. The different data sets were slightly biased, but they were mainly amplified data sets. Survival analysis showed that the survival time of ovarian cancer patients with the TRIB3 mutation was significantly shorter as compared to that of patients without the mutation, indicating that the TRIB3 mutation can predict the survival and prognosis of patients with ovarian cancer.

Tumor immunological destruction is a coordinated multi-step process [36]. Thorsson V [37], performed cluster analysis based on the correlation coefficients between various immune characteristics. All tumors were classified into 6 immune sub-types, among which C3 had the best prognosis. Although sub-types C1 and C2 had many immune components, the prognosis was not good. In sub-types C4 and C6 the immune components were found to be overlapping, providing the worst prognosis. According to the TISIDB database, the expression of TRIB3 was found to be associated with immune sub-types. Level of TRIB3 was found to be higher in sub-types C1, C2, and C4, and lowest in the C3 sub-type. This suggests that the expression of TRIB3 may indicate poor prognosis. CCL7 is a member of the CC chemokine family. Previous studies have shown that CCL7 is secreted by various cells such as tumor cells, fibroblasts, epithelial cells and macrophages. It promoted migration and invasion in prostate cancer, oral squamous cell carcinoma, and gastric cancer [38, 39]. This shows that TRIB3 expression may be involved in regulating the aforementioned immune molecules to promote the progression of ovarian cancer. In additional, TRIB3 was related to the immune-modulation, may not only on itself, but also associated with the MEK/ERK pathway. As for the effects on immunomodulation, the MEK/ERK pathway can played vital roles in B and T cell activation and interleukin signaling pathway [40, 41]. The MEK/ERK inhibitors exerted mild or even stimulatory effects on dendritic cells, CD4+ T cells, and tumor antigen-specific CD8+ T cells[42], but inhibitory effects on infiltrations of regulatory T cells and monocyte/macrophages [43]. In pancreatic cancer, researchers have found that the KrasG12D mutation up-regulated the levels of interleukin-10 (IL-10) and transforming growth factor-β (TGF-β) through activating the MEK/ERK pathway inducing regulatory T cells (Tregs) conversion and immunosuppressive [44]. Furthermore, CCL7 also was regulated by MEK/ERK pathways in many cancers, such as colon cancer cells [45] and Liver metastases [46].

## Conclusions

In summary, this study showed that TRIB3 was significantly over-expressed in ovarian cancer tissues and was associated with poor prognosis of ovarian cancer. It was further proved that TRIB3 over-expression promotes cell proliferation, cell cycle, invasion, migration and EMT of ovarian cancer cells, while inhibiting apoptosis. Mechanistically, TRIB3 may promote the malignant behavior of ovarian cancer by activating the MEK/ERK signaling pathway. With the help of cBioPortal, TCGA, DAVID and Genemania databases, our future studies aim to provide a new dimension for the in-depth research of TRIB3 from the perspective of gene mutation and immunity. We also aim to provide new ideas for further understanding the pathogenesis, diagnosis, and treatment of ovarian cancer.

## Data Availability

The datasets used or analyzed during the current study are available from the corresponding author upon reasonable request.
